# High‐intensity ultrasound applied on cured pork: Sensory and physicochemical characteristics

**DOI:** 10.1002/fsn3.1321

**Published:** 2020-01-15

**Authors:** German Contreras‐Lopez, Andrea Carnero‐Hernandez, Mariana Huerta‐Jimenez, Alma Delia Alarcon‐Rojo, Ivan Garcia‐Galicia, L. M. Carrillo‐López

**Affiliations:** ^1^ Facultad de Zootecnia y Ecología Universidad Autónoma de Chihuahua Chihuahua México; ^2^ Consejo Nacional de Ciencia y Tecnología México City México

**Keywords:** descriptors, drip loss, mass transfer, redness, tenderness

## Abstract

This research aimed to evaluate the physicochemical characteristics and their relationship with sensory properties of cured porcine m. *longissimus lumborum* assisted by high‐intensity ultrasound (HIU, 37 kHz, 22 Wcm^−2^). An experiment was designed with three factors at two levels each: type of curing (immersion or ultrasound‐assisted ‐UA‐), immersion time (30 or 90 min), and steak thickness (1.27 or 2.54 cm). After treatment and 7 days of storage at 4°C, the percentage of salt, pH, CIE L* a* b* color, water holding capacity (WHC), and shear force were determined in the samples. A quantitative descriptive analysis was performed using eight trained panelists. The HIU significantly increased the percentage of NaCl (*p* < .0005) and decreased the color saturation of the meat (*p* < .05), but did not affect the luminosity, redness (a*), yellowness (b*), pH, WHC, or shear force (all *p* > .05). The thickness of the steak had significant effects on almost all of the evaluated variables. Samples with 1.27 cm thickness had lower shear force, higher WHC and salt percentage (*p* < .0001). In agreement with this, the sensory profiles showed that the 1.27 cm samples treated with HIU for 30 min were perceived as less tough (more tender) and juicier.

## Graphical abstract

The application of high‐intensity ultrasound during brining increases the mass transfer (NaCl) without affecting the physicochemical characteristics (color, shear force, and water holding capacity) in pork loins. Thin samples that are ultrasonicated can be perceived as less tough and juicier by consumers.

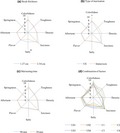

## 1 INTRODUCTION

The curing of meat is one of the oldest methods for its preservation. The use of brine in the meat industry is one of the main technologies for processing meat products, because it improves shelf life, flavor, juiciness, and tenderness (Inguglia, Zhang, Burgess, Kerry, & Tiwari, 2017). The process involves the addition of salts, mainly NaCl and nitrates, which contributes positively to the technological and sensory characteristics of the meat (Ojha, Keenan, Bright, Kerry, & Tiwari, 2016). NaCl is important in processed meat products because it improves technological characteristics, such as water holding capacity, color, fat retention, taste, and texture (Desmond, 2006). Furthermore, NaCl decreases water activity (a_w_) and strongly influences shelf life (Desmond, 2006). There are different commercial salting techniques, such as dry curing, curing with brine, or a combination of both. In both cases, the movement of salt and water out of the meat is dependent on several factors such as type of meat, concentration of salt and time of curing (Ojha et al., 2016). In the commercial industry, a long curing time is necessary due to the complex matrix of meat, which complicates the even distribution of sodium chloride within the muscle (Gou, Comaposada, & Arnau, 2003). Since studies in medical fields have shown that certain plaque deposition inside the coronary arteries may produce atherosclerosis, there is a recent growing interest in the consumption of low salt products (Ruusunen & Puolanne, 2005). However, the reduction of sodium in meat and its products may be inconvenient for preservation. Hence, the evaluation of emerging noninvasive technologies is necessary to guarantee an improvement in salt diffusion inside the muscle matrix, allowing higher uniformity of the ions and its consequent benefit.

Recent studies evaluating HIU used as a curing assisting technology have shown homogeneous ion profiles. For example, González‐González et al. (2017) reported a higher homogeneity of sodium distribution along bovine m. *longissimus dorsi* when marinated with ultrasound assistance for 60 min. There is a growing interest among meat processors to accelerate salt absorption and homogenize profiles in meat (Turhan, Saricaoglu, & Oz, 2013). The interest is to increase organoleptic characteristics, shelf life, and yields. Ultrasound of low frequency and high intensity, also known as power ultrasound, is used in the food industry to accelerate brine processes and improve mass transfer. In meat processing, power ultrasound can modify cell membranes through cavitation, helping to cure, marinate, dry and tenderize the tissue, thereby improving its sensorial and technological quality, and its safety profile (Ozuna, Puig, García‐Pérez, Mulet, & Cárcel, 2013). Moist curing has been combined with HIU as assisted technology, obtaining favorable results. The ultrasound produces bubbles that hit the solids, producing a microinjection of the brine that increases the NaCl content in pork. Further, mass transfer of brine during UA curing of pork depends on the ultrasound intensity (Cárcel, Benedito, Bon, & Mulet, 2007). Siró et al. (2009) also observed that ultrasound (low intensity and low frequency) application caused favorable microstructural changes in pork loin cured with NaCl, which were dependent on intensity. Despite an abundant research describing positive results on technological and sensorial meat quality, there are still many discrepancies caused by intrinsic (specie, age of the animals, aging, and type of muscle) and extrinsic (ultrasound probe, time of exposure, intensity, frequency, temperature, packaging) factors (Alarcón‐Rojo & Janacua‐Vidales, 2017). For example, McDonnell Lyng and Allen (2014) reduced 50% the time for curing ham without any detrimental on quality parameters when ultrasound was used as an assisted technology. On the other hand, Smith (2011) found less water holding without effect on toughness in UA marinated chicken. Ozuna et al. (2013) reported an increment of tenderness in UA marinated pork.

Nevertheless, the effects of ultrasound as assisted technology in curing are not conclusive. This study aimed to evaluate physicochemical characteristics and the relationship with sensory properties of pork loin (*longissimus dorsi*) brining with ultrasound as an assisting technology, considering three factors: muscle thickness, immersion time, and type of marination.

## 2 MATERIALS AND METHODS

### 2.1 Origin of samples and assignment of treatments

Four pork loins (m. *Longissimus lumborum*, ~11 kg, IMPS = 412H, boneless.) were acquired from a regional company in Chihuahua, Mexico. The loins were blast‐frozen (−20°C) after 48 hr *postmortem* which is a common practice in the company. When acquired, they were cut into steaks of either 1.27 or 2.54 cm thickness. For every loin, these two thicknesses were alternated along the loin. Immediately, they were thawed at 4°C for 48 hr, fat and connective tissues were trimmed from them, and steaks were randomly assigned to a treatment, alternating both thicknesses. Original loin was recorded for statistical analysis. Each sample (1.27 or 2.54 cm) was placed in a polyethylene bag (FAB, 20 × 32 cm, 300 caliber) with 0.5 L of brine (2% NaCl). Marination by immersion involved the contact of samples with the brine for 30 or 90 min. In the UA marination, the samples were packed with brine and were ultrasonicated (37 kHz and 22 Wcm^−2^) in a bath (Elmasonic® S60H). Distilled water at 4°C was used as a coupling medium. This gave a total of eight treatments (thickness × marinating time × ultrasound) with three repetitions each, obtaining a total of 24 samples for the physicochemical analysis and 24 for the sensory analysis. After marination (immersion or UA), the samples were vacuum‐packed (polyethylene bags 70 caliber, processed in a Koch ®, Easypack ™ vacuum packer) and stored for 7 days at 4ºC and later they were unpacked for analyses of the variables.

### 2.2 Determination of salt

The salt concentration in the muscle was determined in triplicate with a digital salinometer (ES‐421), based on the conductivity method (g of NaCl/ 100 g of muscle). Ten grams of sample were macerated in 90 ml of distilled water and placed in the salinometer. The value obtained with the equipment was multiplied by 10 to obtain the percentage of salt.

### 2.3 Determination of pH

pH was measured in triplicate with a digital puncture pHmeter (Sentron®, Model 1001). The introduction of the electrode into the muscle was performed perpendicular to the sample at 2 cm depth, avoiding contact with remaining fat and connective tissue (Honikel, 1998).

### 2.4 Color analysis

The CIE L*a*b* color parameters were obtained with a colorimeter (Konica Minolta, CR 400). The measurement was carried out under the CIE (Commission Internationale Pour L’Eclarige) reference system, according to the methodology of the AMSA (2012). The samples were allowed to oxygenate for 20 min. Three readings were made per sample, registering the values L*, a*, and b*. Chroma (C*) was calculated by means of the expression: C* = √a*^2^ + b*^2^.

### 2.5 Water holding capacity (WHC)

WHC was determined by the compression method proposed by Tsai and Ockerman (1981). 0.3 g of ground pork were weighted using an analytical balance (±0.05 g) and located between two layers of filter paper #1 (Whatman^®^), and this between two methacrylate plates. A constant weight of 10 kg was located on the sample for 20 min. WHC was expressed as percent difference in sample weight before and after pressure application according to the following equation: water retention capacity (%) = (100‐ free water), where: free water = (final weight of the filter paper − initial weight of the filter paper)/ sample weight × 100.

### 2.6 Determination of shear force

Shear force was evaluated according to the methodology of the AMSA (American Meat Science Association) (2015). The samples were placed in commercial plastic bags (to avoid exudate being released into bath) and cooked in a water bath (Isotemp® 215, 85°C) until reaching 72ºC in the geometric center (from 10 to 12 min aprox.). Temperature was monitored by a thermocouple attached to an infrared digital thermometer (Fisherbrand™ Traceable™ Infrared Thermometer with Trigger Grip). After cooking, excess water was drained from the bag, and samples were stored at 4ºC for 24 hr. Then, nine cylinders of 1.27 cm in diameter were obtained (parallel to the muscle fibers) from each sample. The cylinders were cut perpendicular to the direction of the muscle fibers using the Warner‐Bratzler V‐blade (Stable Micro Systems Ltd.). Parameters established for the test were speed of 2 mm/s and distance of 30 mm. Maximum peak force was recorded during the test and expressed in kgf.

### 2.7 Quantitative descriptive analysis and preference test by ordering

A quantitative descriptive analysis (QDA) was performed. A group of eight panelists were trained according to the procedures of Meilgaard, (2006) and AMSA (American Meat Science Association) (2015). Evaluated descriptors were as follows: colorfulness, toughness, density, springiness, juiciness, salt, flavor, and aftertaste. The definition of the sensory attributes, as agreed and discussed by the panelists, is shown in Table 1. The panel consisted of four women and four men between 24 and 44 years old. All of them were frequent consumers of pork (three times/week). The evaluations were made in individual cubicles of the Sensorial Analysis Laboratory of the University of Chihuahua, using red filters to mask shades. The laboratory temperature was controlled at 20°C and the illumination was white fluorescent. The cooking was carried out on electric plates (George Foreman^®^). Samples were cooked at 72ºC (geometric center). Temperature was monitored with a thermocouple connected to an infrared digital thermometer (Fisherbrand™ Traceable™ Infrared Thermometer with Trigger Grip) and served immediately in glass dishes. The sample temperature was 35°C when offered to panelists. Distilled water was provided to panelists for rinsing their mouth between samples. A unstructured scale of 15 cm with anchors of 1.5 cm was used on each side for the QDA.

**Table 1:** Description of the sensory attributes used in the quantitative descriptive analysis of pork loin

Characteristic	Attribute	Definition
Appearance	Colorfulness	Purity of color on the surface of the meat (0 = opaque, 15 = shiny)
Texture	Toughness	Strength required to achieve muscle deformation (0 = soft, 15 = hard)
	Density	Compactness of the cross section (0 = light, 15 = heavy)
	Springiness	Speed of return to the original form after deformation (0 = little, 15 = much)
	Juiciness	Release of juices (water/ fat) during chewing (0 = little, 15 = a lot)
Flavor	Salt	Basic taste stimulated by sodium salts (0 = little, 15 = a lot)
	Flavor	The combined effect of taste sensations, aromatics, and chemical feeling factors evoked by a substance in the oral cavity (0 = little, 15 = much)
	Aftertaste	The oral or nasal sensations that occur after the stimulus have been removed from the oral cavity (0 = little, 15 = much)

### 2.8 Statistical analysis

The data were analyzed in a factorial design where the experimental unit was the pork sample. Curing type (immersion vs. assisted by ultrasound) × immersion times (30 vs. 90 min)  × muscle thickness (1.27 vs. 2.5 cm) were included in the model as factors. When significant differences were detected (*p* < .05), a Tukey test was performed to compare means using *p* < .05. For all QDA data, analyses of variance, factor analyses, and an analysis of major components was performed. Data of the test based on ranks were analyzed based on a comparison of all the samples (treatments) among themselves. The multiple comparison procedure involved the addition of Friedman ranges for the analysis of ordinal data. Data analysis was performed in the statistical package SAS v. 9.4.

## 3 RESULTS AND DISCUSSION

### 3.1 Salt content in meat

**Figure 1:**
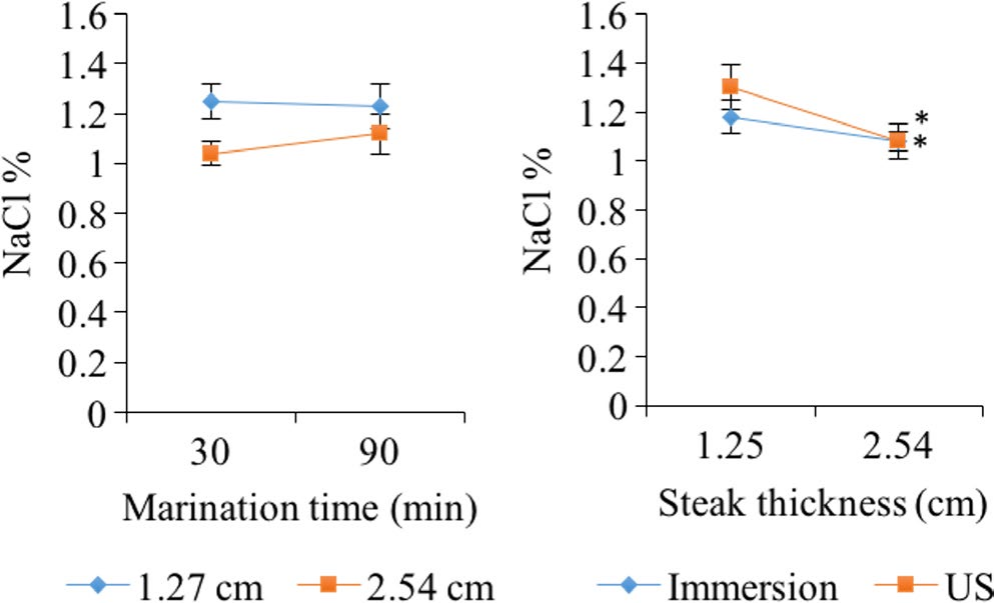
Effects of the marinating type (immersion or ultrasound‐assisted), marinating time (min), and steak thickness of m. *longissimus* (cm) on the percentage of salt in pork. Asterisks (^*^) indicate significant differences (*p* < .05)

Significant differences were found in the salt content of the meat related to steak thickness (*p* < .0001) and marinating type (*p* = .0005). Thinner steaks (1.27 cm) had a higher NaCl content (Figure 1), independent of the marinating time (30 vs. 90 min). Steaks of 1.27 cm thickness with US‐assisted marination had a higher percentage of NaCl (Figure 1). However, in steaks of 2.54 cm thickness, no differences were found between marinating methods. Possibly, the 1.27 cm samples showed a higher salt content due to the physical characteristics of the area/volume ratio, which promoted a higher NaCl transfer. Regarding the US application, Ojha et al. (2016), Siró et al. (2009), and Ozuna et al. (2013) established that the phenomenon of cavitation by US induces a microagitation phenomenon, forming microchannels that increase the NaCl transfer to the muscle. Short marinating times are sufficient for a significant increase of NaCl content in lean muscles (1.27 cm). However, for thicker muscles (2.54 cm), it may be necessary to increase the sonication time. It is also possible that higher ultrasonic intensities could have a higher effect on the mass distribution inside the tissue. In this regard, McDonnell Lyng Morin and Allen (2014) found that US application during meat curing increases the absorption of brine. However, intensities of 19 W/cm^−2^ for 25 min were required to increase the NaCl content in meat. González‐González et al. (2017) found that during the US‐assisted marination the sodium distribution was more homogeneous and its mass transfer dependent on the storage time and the type of marinade. Hence, longer storage times (7 days, at 4ºC) increase the amount of sodium (%) in bovine *longissimus dorsi*. On the other hand, Goli, Bohuon, Ricci, Trystram, and Collignan (2011) found that solute infiltration is dependent on the concentration of NaCl in brine. In this case, saturated solutions decrease WHC and increase attraction forces among myofibrillar proteins. This effect is due to the decrease in pH in the presence of NaCl, which causes contraction of the matrix, especially during long marinating times (16 min). Supporting the results reported by Goli et al. (2011), the thickness of the muscle is also an important factor that influences the degree of solute transfer from the marinade solution to the muscle (Figure 1). Thus, the literature indicates that long marinating times may be justified in thick pieces of muscle.

### 3.2 Color

The luminosity (L*) of lean was not affected by the effect of muscle thickness, time of marination or type of marination (*p* ˃ .05; Table 2). In general, moderate high values of L* (52–56) indicate a higher tendency toward clear colors, which may favor the intention of purchase by consumers who dislike very pale or dark pork colors. In contrast to the results in this study, Gómez‐Salazar, Ochoa‐Montes, Cerón‐García, Ozuna, and Sosa‐Morales (2018) found a significant increase in the values of L* in rabbit meat with US‐assisted marinating (ultrasonic bath, 40 kHz, 100 W), when using a combination of NaCl, NaNO_3_ and citric acid in marinades. L* values reported in the present study are normal for pork. For instance, Arowolo et al. (2019) found values from 51.06 to 52.51, depending on the diet of the pigs.

**Table 2:** Effect of marinating type (immersion or high‐intensity ultrasound‐assisted), marinating time (min), and steak thickness (cm) on CIE color parameters L*, a*, and b* of pork loins

Variable	CIE L*a*b*			
Thickness (cm)	L*	a*	b*	Saturation
1.27	56.34 ± 0.88^a^	3.37 ± 0.74^b^	5.99 ± 0.38^b^	6.9 ± 0.62^b^
2.54	56.22 ± 1.94^a^	5.29 ± 0.88^a^	7.77 ± 1.28^a^	9.41 ± 1.55^a^
Marination time (min)	L*	a*	b*	Saturation
30	56.46 ± 1.38^a^	4.69 ± 1.19^a^	7.62 ± 1.52^a^	8.65 ± 1.88^a^
90	56.1 ± 1.6^a^	3.96 ± 1.27^b^	6.5 ± 0.92^b^	7.65 ± 1.41^b^

Marination type	L*	a*	b*	Saturation
Ultrasound‐assisted	56.53 ± 1.41^a^	4.04 ± 1.17^a^	6.8 ± 1.26^a^	7.93 ± 1.63^a^
Immersion	56.03 ± 1.56^a^	4.61 ± 1.34^a^	6.96 ± 1.37^a^	8.37 ± 1.82^b^

Note

Column means within a factor with different superscripts differ significantly at *p* < .05.

Statistical differences were found in redness (a*) and yellowness (b*), related to the effect of steak thickness (*p* < .0001) and marinating time (*p* = .0295) (Table 2). Thick muscles (2.54 cm) and those marinated for short times (30 min) showed a higher redness and yellowness. Myoglobin is the main compound providing the color in the meat. When meat is in contact with water or other solutions (immersion), myoglobin undergoes dilution, since it is a water soluble protein. This explains the low values in a*. In addition, thin samples and samples that were marinated for short times presented lower values of a*, because the myoglobin could be diluted more quickly in thin samples, facilitating the rapid mass transfer to the interior. In bovine meat, a more pronounced change in color parameters of samples marinated by immersion and assisted with US has been observed. Jayasooriya, Torley, D’Arcy, and Bhandari (2007) established that US alone does not affect the color parameters; however, the ripening time significantly increases brightness, chroma, and tone. On the other hand, Gómez‐Salazar et al. (2018) reported a significant decrease in rabbit meat a* values when the NaCl concentration was increased and with the application of US. The same authors reported an increase in b* values when rabbit meat was marinated by US treatment with 70 and 140 g of NaCl/ L. Pohlman, Dikeman, and Kropf (1997) found modifications in color parameters of ultrasonicated m. *Pectoralis* (22 Wcm^−2^, 20 kHz), increasing values of b* and L* and decreasing a*. Those results are in contrast to the results reported in this study.

Regarding chroma or color saturation, significant differences were found by effect of steak thickness (*p* < .0001) and marination time (*p* = .0399) (Table 2). Visually, saturation of color indicates the intensity, hence, high values indicate higher brightness and low values higher opacity or absence of color. Consistently with the results of a* and b*, thicker samples (2.54 cm) presented higher values of chroma, a reason for a more shining perception. Furthermore, treatments with short marinating times (30 min) showed higher color saturation. Short marinating times result in higher mass transfer, which visually causes a perception of a higher surface vividness. No color changes were found by effect of US. This agrees with McDonnell, Lyng, Morin, et al. (2014), who did not find changes in the values of L*, a*, and b* in cured and US‐assisted pork (10, 25 and 40 min; 4.2, 11 and 19 Wcm^−2^). Conversely, other researchers have observed changes in color parameters of bovine meat immersed in distilled water (Chang, Xu, Zhou, Li, & Huang, 2012; Pohlman et al., 1997). It is well known that acoustic cavitation may generate oxidation of biomolecules such as lipids and proteins. For instance, Kang et al. (2016) showed that ultrasonication (20 kHz, 2.39–20.96 Wcm^−2^) of beef (48 hr *postmortem*) in brine (6% NaCl) increased oxidation of lipids and proteins. Additionally, US causes changes in free sulfhydryl groups and protein hydrophobicity, which modifies the secondary structure by an increase in β‐sheet and a decrease in α‐helix (Kang et al., 2016). During ultrasonication, chemical reactions can cause hydrolysis and oxidation of lipids, generating reactive oxygen species that modify and oxidize intracellular and membrane proteins in the muscle (Kang et al., 2016). Chemical changes include aggregation, cross‐linking, degradation, and fragmentation of proteins, depending on the nature of the protein component and the free radicals that attack (Gómez‐Salazar et al., 2018). This may lead to the alteration of enzymatic activity, and cellular and membrane functioning (Wolff, Garner, & Dean, 1986). Changes associated with color parameters were reported by Pohlman et al. (1997), who found pale meat samples, with orange tones and less lightness due to denaturation of myoglobin by high temperatures in the US device (22 Wcm^−2^, 5–10 min).

### 3.3 pH

**Figure 2:**
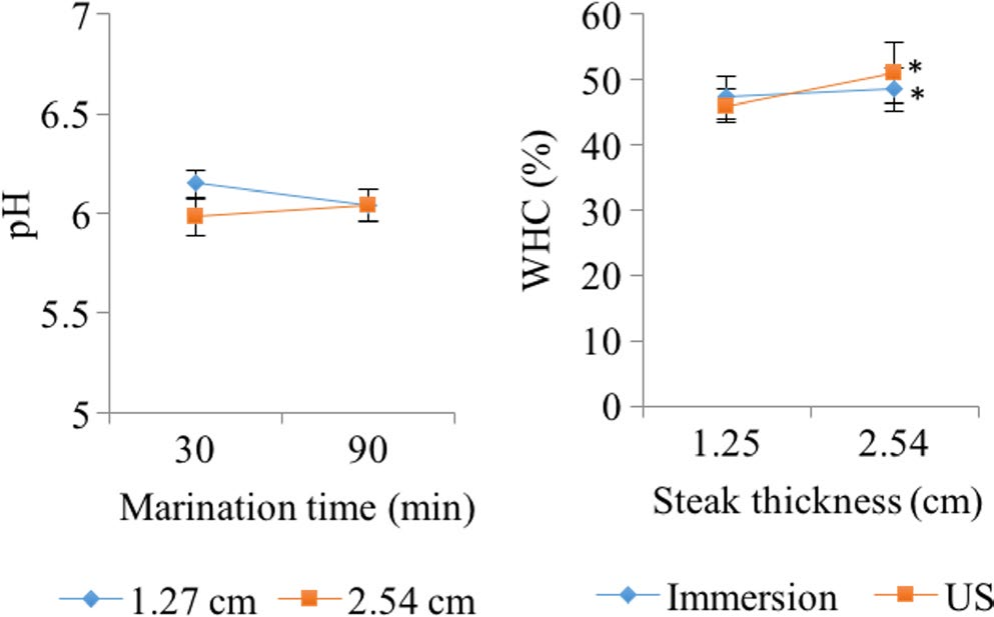
Effect of the type of marination (immersion or high‐intensity ultrasound‐assisted, US), marination time (min) and steak thickness (cm) on pH and water holding capacity (WHC, %) of pork loins. Asterisks (^*^) indicate significant differences (*p* < .05)

Significant differences were observed in muscle pH (Figure 2) by differences of steak thickness (*p* < .0001). There was no difference in pH by effect of time (*p* = .237) or type of marination (*p* = .103). However, there was a significant interaction of steak thickness × marinating time (*p* < .0001). After 30 min of marination, the pH of 1.27 cm thick samples was significantly higher than those of 2.54 cm (Figure 2). Possibly, this effect was due to the increase in the salt and water content (Figure 1). In contrast, after 90 min of marination, the pH of both samples (1.27 and 2.54 cm) was similar. An increase in pH by ultrasonication is due to the release of ions from the cellular structure to the cytosol and to the change in the structure of the protein (Alarcon‐Rojo et al., 2018). This leads to a modification in the position of some ionic groups, which makes them available for a muscle buffer reaction (Gambuteanu, Filimon, & Alexe, 2013). According to Got et al. (1999), when muscles have a fast pH drop range, the buffering capacity induced by the US could be too small to show significant differences between treatments, which supports the results of this study, with no effect of the US on muscle pH. Reports on the influence of ultrasound on pH are inconclusive. McDonnell, Lyng, Morin, et al. (2014) found no significant effect on pH of cured pork due to ultrasonication (10, 25, and 40 min; 4.2, 11 and 19 Wcm^−2^). Jayasooriya and et al., (2007) reported a significant increase in pH in bovine *Longissimus lumborum et thoracis* and *Semitendinosus* by application of US (24 kHz, 12 Wcm^−2^, 240 s) and aging time (8.5 days).

### 3.4 Water holding capacity (WHC)

WHC was not affected by marinating time (*p* = .567) or type of marination (*p* = .4766) (Figure 2); however, thicker samples (2.54 cm) had higher WHC than thin samples (1.25 cm) (*p* = .0004). This difference was possibly due to a higher exposed surface area of the thick steaks, where the area/volume ratio resulted in an increase in its capacity to absorb water and/or solutes. It is well known that salts are strong water retainers; for instance, Kenney and Hunt (1990) reported that four percent of NaCl produced a higher protein dissolution and water holding capacity in preblended meat. Some studies report that ultrasound‐assisted immersion results in significant increases of WHC (McDonnell, Lyng, Morin, et al., 2014; Siró et al., 2009). Although the ultrasonication did not have a significant effect by itself, the interaction of the steak thickness × type of marination (US‐assisted or immersion) was significant (Figure 2), with WHC values of 51% in thick ultrasonicated samples. Our results are similar to those obtained by Siró et al. (2009), who reported that US treatment improved WHC in pork ultrasonicated with 2.5 Wcm^‐2^ for 180 min. In this case, low US intensities (2 and 2.5 Wcm^−2^) require long US times to increase the WHC, while high US intensities (3 and 4 Wcm^−2^) decrease the WHC due to protein denaturation. Goli et al. (2011) reported high densities of solute flow in cubes of turkey meat (1 cm^3^) marinated with low concentrations of NaCl and acetic acid, due to the expansion of the protein matrix (myofibrils). However, high concentrations of NaCl cause dehydration in muscle, regardless of the acid content. In agreement with the results of Goli et al. (2011), the thickness of the tissue constitutes another important factor during the marination process, where thick samples reach higher levels of WHC due to the higher expansion of the protein matrix after 7 days of aging at 4ºC. Biochemically, NaCl has an effect on the properties of holding and retention of water for the muscle, due to the swelling of myofibrillar proteins. Formation of an ionic cloud of sodium around muscle filaments leads to an increase in osmotic pressure within myofibrils, with the consequent swelling that provides an upper number of protein side chains binding water (Cheng & Sun, 2008).

### 3.5 Shear force

**Figure 3:**
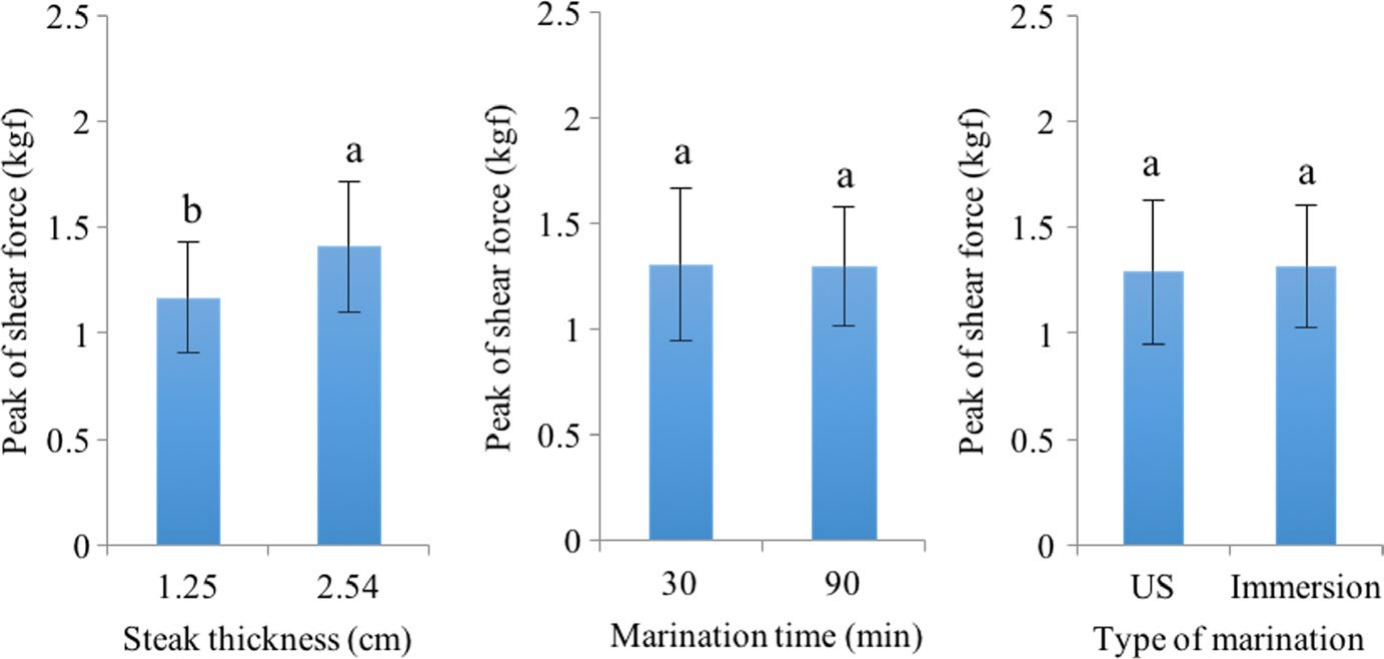
Effect of the marinating type (immersion or high‐intensity ultrasound‐assisted), marinating time (min), and steak thickness (cm) on peak of shear force (kg) of pork loin. Means with different superscripts differ significantly at *p* < .05

No statistical differences were found in shear force (Figure 3) by marinating time (*p* = .6343) or type of marination (*p* = .4514). However, thicker steaks had a significant higher shear force (*p* < .0001). Interactions between factors were not significant (*p* > .05). Ozuna et al. (2013) found that content of NaCl increases the shear force of pork loins, while the use of high‐intensity ultrasound increases the transfer of solutes in tissue. In this study, there was no change in tenderness of pork loin by high‐intensity ultrasonication, despite a higher mass transfer in ultrasonicated samples. Concentrations of NaCl in brine for this study were lower than those used in other studies (Ozuna et al., 2013; Siró et al., 2009), with concentrations from 4 to 5 to 28 add unit of measurement. Possibly, the amount of NaCl in the brine used in this study was not enough to observe changes in the tenderness of the meat. In this regard, Siró et al. (2009) found a decrease in tenderness of ultrasonicated pork (2.5 and 3 Wcm^−2^), caused by the destructive nature of acoustic cavitation and the vibration of the ultrasonic waves that cause the weakening of the structure (Jayasooriya et al., 2007). However, high US intensities may have negative effects because they potentially cause protein denaturation and low WHC, increasing muscle toughness. In regard to steak thickness, Panea, Sañudo, Olleta, and Civit (2008) reported similar results to those of the present study, demonstrating that from different thicknesses (1, 2, and 4 cm) of bovine muscle, the thickest had the highest shear force. On the other hand, Goli et al. (2011) reported that because of the porous nature of the meat, the protein network facilitates the infiltration of solutes during marinating. The fibers are separated by losing part of their integrity during *postmortem* aging.

### 3.6 Sensory analysis

**Figure 4:**
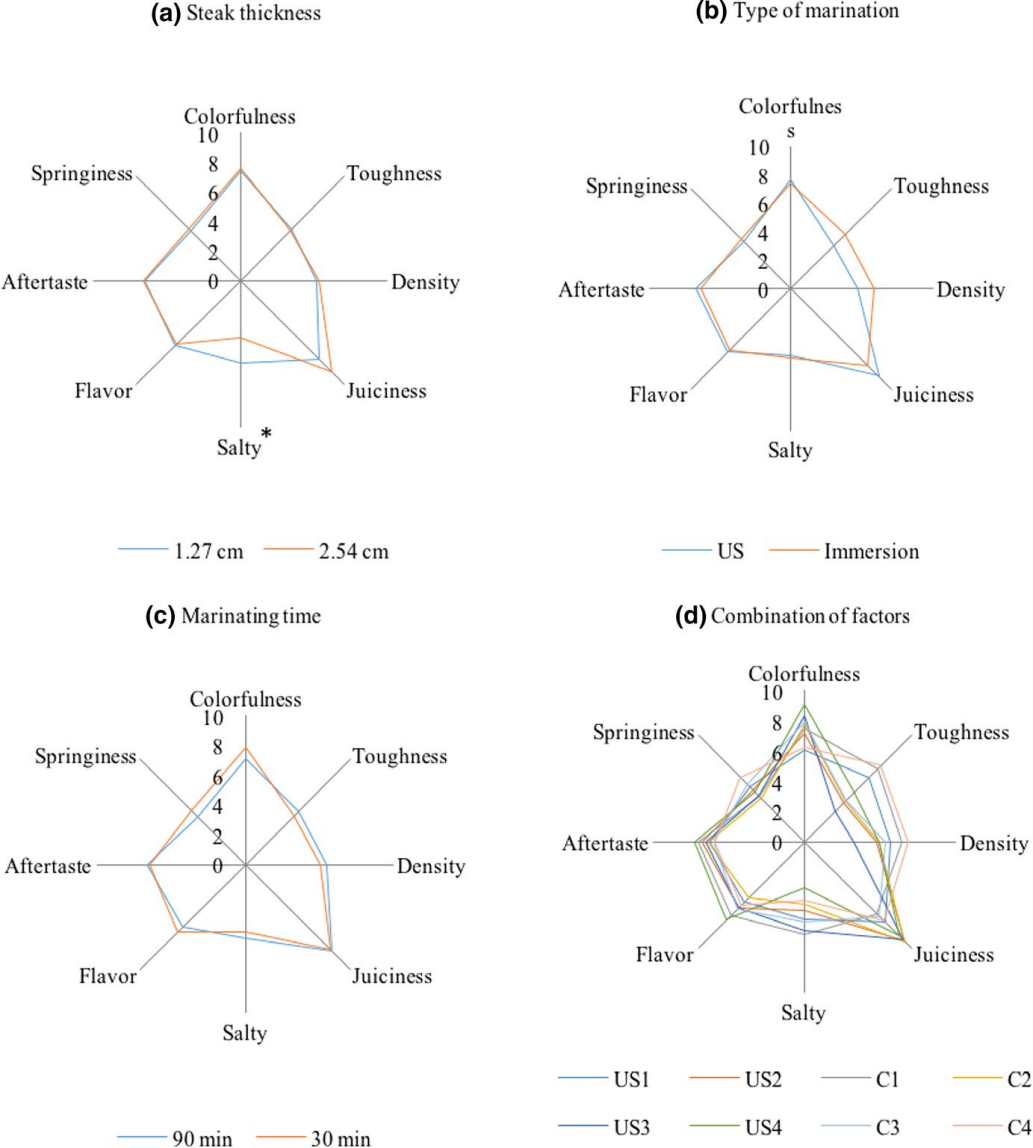
Sensory descriptors in pork loins marinated by immersion or assisted by high‐intensity ultrasound (US: 37 kHz, 22 W * cm^−2^), after storage at 4ºC for 7 days. Effects of (a) steak thickness, (b) type of marination, (c) marinating time, and (d) combination of factors. US1: US 90 min 1.27 cm; US2: US 90 min 2.54 cm; US3: US 30 min 1.27 cm; US4: US 30 min 2.54 cm; C1: Immersion 90 min 1.27 cm; C2: Immersion 90 min 2.54 cm; C3: Immersion 30 min 1.27 cm; C4: Immersion 30 min 2.54 cm. Asterisks (^*^) indicate significant differences (*p* < .05)

Sensory profiles were performed considering the effect of individual factors: steak thickness (Figure 4a), type of marination (Figure 4b) and marinating time (Figure 4c), and the combination of these factors (Figure 4d). Significant differences were found between thick and thin samples only for the salty; thin samples were perceived saltier by panelists (*p* = .0227). Other sensory attributes did not show significant differences between steak thickness, although a higher juiciness was observed in 2.54 cm samples. This is positively related to WHC and % salt, where 1.27 cm samples showed higher mass transfer (% salt). Further, 2.54 cm thick samples had a higher WHC and, consequently, they were perceived as juicier by the panelists.

Regarding the type of marination (US‐assisted or immersion), no statistical differences were found (*p* > .05). McDonnell, Lyng, Morin, et al. (2014) found that ultrasonicating pork while curing had a potential positive effect on texture, decreasing cohesiveness (binding strength within the food), toughness (strength during the first compression), and gumminess (toughness × cohesiveness), without any detrimental effect on other quality parameters. In this regard, the density may be equivalent to the cohesiveness measured objectively by the analysis of the texture profile. Hence, our results are analogous to those obtained by McDonnell, Lyng, and Allen (2014), in terms of the decrease in density in US‐assisted cured samples. Similar to the instrumental analysis, there were no differences in shear force and WHC between type of marination (immersion or US‐assisted). In other studies, such as González‐González et al. (2017), no significant differences in the acceptance of marinated beef by US or immersion are reported. However, the study was carried out with consumers and the authors recommended a descriptive analysis with trained panelists. Results similar to ours were obtained with Peña‐González et al. (2017), who reported that samples with US (40 kHz, 11 Wcm^−2^) during 60 min were perceived by trained panelists as tender and juicier than controls after 14 days of storage at 4ºC.

Unexpectedly, marination time had no effect on the perception of the evaluated attributes (Figure 4c), whereas when combined treatments were analyzed (Figure 4d), samples of 1.27 cm thickness and ultrasonicated for 30 min were perceived as the less tough and juicier. A wider range of treatment means made evident that salty, toughness, colorfulness, and juiciness were the attributes with the highest variability among treatments. Correlating the sensory with the instrumental analysis, samples of 1.27 cm thickness (both sonicated or immersed) had a higher WHC and lower shear force and were perceived as juicier and less tough by panelists. In this regard, Cárcel et al. (2007) reported that high‐intensity ultrasound fields microinjected the brine into the meat, leading to a direct increase of NaCl and water in the tissue.

**Figure 5:**
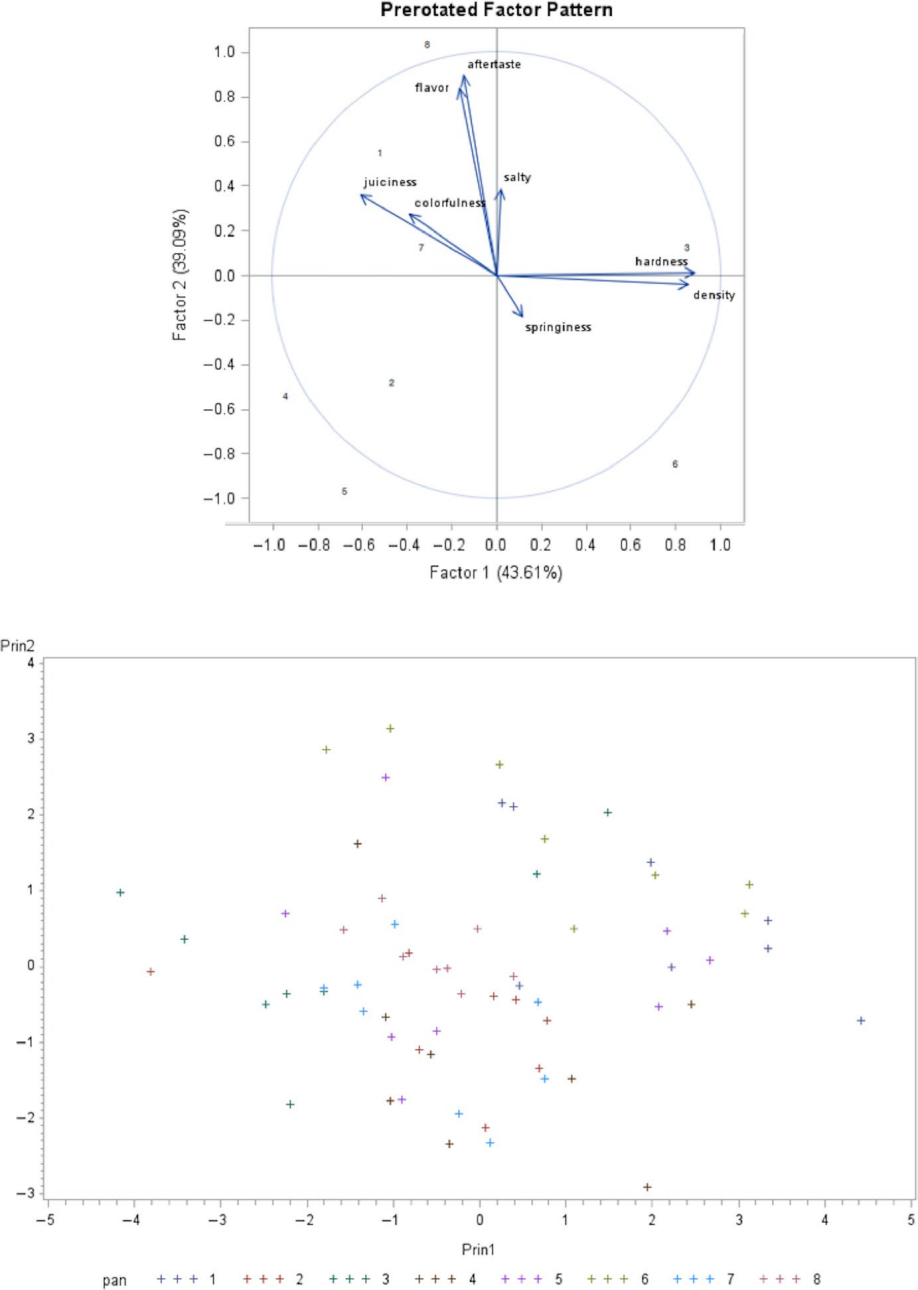
Two‐dimensional projection by factorial analysis and principal components for eight treatments of pork loin: (1) US 90 min 1.27 cm; (2) US 90 min 2.54 cm; (3) Immersion 90 min 1.27 cm; (4) Immersion 90 min 2.54 cm; (5) US 30 min 1.27 cm; (6) US 30 min 2.54 cm; (7) Immersion 30 min 1.27 cm; (8) Immersion 30 min 2.54 cm; pan = panelist

The results of the principal component analysis showed that 43.61% of the total information is represented by the first factor, and 39.09% by the second factor, together explaining 82.7% of the total variation of the data (Figure 5). The remaining 27.3% can be discarded to observe the data in two dimensions (Figure 5). Measurements close to each other correlate positively, whereas measurements separated by 180º are negatively correlated. If they are separated 90º, they are independent (Cañeque et al., 2004). The longest vectors are the most important descriptors and correspond to toughness, density, aftertaste, flavor, and juiciness, while the least important attributes were salty, colorfulness, and springiness. Aftertaste and flavor descriptors present a good interrelation; flavor is released into the oral cavity during the process of mastication, which is positively related to the aftertaste after swallowing. Colorfulness is projected in the same direction of juiciness because both are directly related to WHC. Samples with higher WHC will have higher juiciness and moisture on the surface, so they are perceived visually with more brightness (higher colorfulness, chroma, intensity, or purity). Appearance is one of the most important characteristics in meat acceptance by consumers, and often determines the purchase intention (De Huidobro, Miguel, Blázquez, & Onega, 2005). Finally, on the bidimensional projection (Figure 5), it is evident that the immersed meat (treatment 7, 30 min, 1.27 cm) was defined by juiciness descriptor (in Figure 4d, was observed as the less juicy), meanwhile the group of samples 1 (US‐assisted for 90 min, 1.27 cm) was particularly defined by colorfulness descriptor (identified by a higher opacity in Figure 4d). The treatment 8 is projected in the same direction than aromatic and aftertaste descriptors, being defined by low levels of those characteristics.

In the two‐dimensional chart, it is also observed that density and toughness are closely related. In this sense, denser samples have less air space (volume) and tend to be harder and heavier. Springiness was not related to any descriptor.

Few studies have reported a sensory evaluation of samples that were ultrasonicated during marinating. In this regard, data analysis included ANOVA presented in radial graphs that include sensory attributes (González‐González et al. (2017), Peña‐González et al., 2017). However, a multivariate analysis of sensory attributes may be a more powerful technique that allows to reduce attributes and explain correlations among them inside factors. In this regard, Mwove, Gogo, Chikamai, Omwamba, and Mahungu (2018) studied the relationship among 24 measurements of beef quality injected with brine containing arabic gum and soy protein concentrate under two injection levels (30 and 35%). They found that juiciness and higher levels of injection in meat defined the general acceptance of the product. Regarding the evaluation of the panelists (Figure 5), we observed the formation of a cloud in the center of the graph, which indicates consistency in the panelists during the analysis of the samples. However, the dispersion observed for panelists 1, 5, and 6 in the bidimensional chart of the main components indicates that they might need a longer training. Peña‐González, Alarcon‐Rojo, Garcia‐Galicia, Carrillo‐Lopez, and Huerta‐Jimenez (2018)reported that meat stored for 14 days and then treated with ultrasound (40 kHz, 11 Wcm^−2^) was perceived to have a more intense fresh meat smell and oily flavor; however, it was also perceived to be a paler grayish brown color compared to control samples stored for the same duration; ultrasonicated meat also presented a greater intensity of metallic taste and a less tough and moist texture. This effect of ultrasound was not observed in the present study, where ultrasonicated samples were perceived as juicier and less tough by panelists.

## 4 CONCLUSIONS

The application of high‐intensity ultrasound during brining significantly increased the NaCl percentage (*p* < .0005) and decreased the color saturation in pork loins, but it did not affect the lightness or a* and b*, nor did it modify the pH, shear force, or water holding capacity (WHC). Steak thickness and marinating time significantly influenced the physicochemical quality and sensory perception of marinated meat. Thin samples (1.27 cm) had a lower shear force, higher WHC, and salt content than thicker samples (2.54 cm). However, lightness, redness, and yellowness decreased strongly. On the other hand, long marination times (90 min), negatively influenced the pork quality, reducing redness, yellowness, and lightness of the meat. In sensory tests, panelists perceived the 1.27 cm samples as saltier, while no significant differences for the other sensory attributes were described. Apparently, samples of 1.27 cm thickness that were ultrasonicated for 30 min during marination were perceived as less tough and juicier.

## Conflict of Interest

The authors declare that they have no conflict of interest.

## AUTHOR CONTRIBUTIONS

Luis M. Carrillo‐Lopez designed the experimental study and drafted the research article. German Contreras‐Lopez and Andrea Carnero‐Hernandez helped obtaining experimental data. Finally, Mariana Huerta‐Jimenez, Alma D. Alarcon‐Rojo, and Ivan A. Garcia‐Galicia helped with the editing of the manuscript.

## ETHICAL STATEMENTS

The authors state that human and vertebrate animal testing was unnecessary in this study. Informed Consent: Written informed consent was obtained from all study participants.
